# Different Phenotypes of Sarcomeric *MyBPC3*-Cardiomyopathy in the Same Family: Hypertrophic, Left Ventricular Noncompaction and Restrictive Phenotypes (in Association with Sarcoidosis)

**DOI:** 10.3390/genes13081344

**Published:** 2022-07-27

**Authors:** Olga Blagova, Ekaterina Pavlenko, Vsevolod Sedov, Evgeniya Kogan, Margarita Polyak, Elena Zaklyazminskaya, Yulia Lutokhina

**Affiliations:** 1 I.M. Sechenov First Moscow State Medical University (Sechenov University), 119435 Moscow, Russia; blagovao@mail.ru (O.B.); evd88@mail.ru (E.P.); vps52@mail.ru (V.S.); koganevg@gmail.com (E.K.); 2 B.V. Petrovsky Russian Scientific Center of Surgery, 119991 Moscow, Russia; ametane@yandex.ru (M.P.); helenezak@gmail.com (E.Z.)

**Keywords:** hypertrophic cardiomyopathy, restrictive cardiomyopathy, left ventricular noncompaction, sarcoidosis, *MyBPC3*, truncating pathogenic variants

## Abstract

The same variants in sarcomeric genes can lead to different cardiomyopathies within the same family. This gave rise to the concept of a continuum of sarcomeric cardiomyopathies. However, the manifestations and evolution of these cardiomyopathies in pathogenic variant carriers, including members of the same family, remains poorly understood. We present a case of familial sarcomeric cardiomyopathy caused by heterozygous truncating pathogenic variant p.Q1233* in cardiac myosin-binding protein C (*MyBPC3*) gene. The proband was first diagnosed with restrictive cardiomyopathy combined with left ventricular noncompaction (LVNC) and sarcoidosis at the age of 64. The predominantly restrictive phenotype of cardiomyopathy is considered to be a result of interaction between LVNC and sarcoid myocarditis. His 39-year-old son and 35-year-old daughter have identical non-obstructive asymmetric hypertrophic cardiomyopathy. The risk of sudden cardiac death in the son is high due to myocardial fibrosis, ischemia and nonsustained VT. We assume that both phenotypes in the family may have originally been different or there may have been a gradual transformation of the hypertrophic phenotype into LVNC. Myocarditis is regarded as an important epigenomic modifier of sarcomeric cardiomyopathy. In the proband and his son, cardioverter-defibrillators were implanted, and the proband experienced appropriate shocks due to ventricular tachycardia/fibrillation. The proband was also treated with corticosteroids. His death at the age of 69 years occurred due to acute gastric hemorrhage accompanied by progressive heart failure. This report confirms the concept of the phenotypic continuum of sarcomeric cardiomyopathies and describes possible phenotypic patterns and their transformation over time.

## 1. Introduction

There is a pronounced genetic heterogeneity of primary cardiomyopathies, making it relevant to study the role of various genetic changes in the formation of different phenotypes. Hypertrophic cardiomyopathy (HCM) is the morphofunctional variant of cardiomyopathy for which the genetic basis is best understood. HCM is caused by pathogenic variants in the genes encoding sarcomeric proteins. About half of identified pathogenic variants were found in the *MyBPC3* gene [1]. Pathogenic and likely pathogenic variants in the *MyBPC3* gene are the most common cause of HCM, and about 90% of the known mutations in this gene result in premature termination codons [2]. However, the same variants in sarcomeric genes are described in patients with different phenotypes of cardiomyopathies: hypertrophic, restrictive and left ventricular noncompaction (LVNC) [3,4].

The red flags of HCM in addition to the basic criteria (a wall thickness ≥15 mm in one or more myocardial segments—as measured by any imaging technique) are family history, absence of any extra-cardiac symptoms and signs, electrocardiogram abnormalities, laboratory tests and multi-modality cardiac imaging. The clinical diagnosis of HCM in first-degree relatives of patients is based on the presence of unexplained increased LV wall thickness ≥13 mm in one or more LV myocardial segments.

The nature of LVNC remains much less certain. This is the only type of cardiomyopathy for which consensus guidelines or a position statement are still absent. Heterogeneous phenotypes of LVNC, ranging from asymptomatic and arrhythmic to dilated, restrictive, and hypertrophic, have been reported in children and adults [5,6]. The genetic defects associated with LVNC were found in the wide range of genes encoding sarcomeric structural mitochondrial proteins and subunits of cardiac ion channels [7,8]. There are numerous reports of LVNC combinations with more clearly defined types of cardiomyopathies, in particular, hypertrophic [9,10,11].

On the one hand, these data challenge the independence of LVNC as a special cardiomyopathy. On the other hand, they suggest the concept of a continuum of sarcomeric cardiomyopathies, which emphasizes their pathogenetic unity and possibility of the mutual transformation [12]. Cases of different phenotypic manifestations of sarcomeric cardiomyopathy within the same family are unique and most clearly illustrate this hypothesis [13,14]. The mechanism of transformation of different phenotypes of cardiomyopathy in one patient remains unclear and requires further investigation. Myocarditis is considered to be an important epigenomic factor that affects the phenotypic manifestations of sarcomeric mutations. However, there are no reports of a similar clinical scenario, which makes the present case of familial cardiomyopathy interesting and unique.

## 2. Materials and Methods

The proband, a 64-year-old male non-smoker, was first admitted to the clinic in June 2014 with complaints of breathlessness during low physical activity, leg edema, infrequent palpitations, occasional pulling pains in the cardiac area and a tendency to hypotension (blood pressure (BP) 100/60 mm Hg).

The patient underwent physical examination and clinical evaluation including collection of venous blood samples for DNA diagnostics, blood tests (full blood count, biochemical panel and anti-heart antibodies), electrocardiography (ECG), 24-h ECG monitoring, ultrasound cardiography (UCG), coronary angiogram, cardiac magnetic resonance imaging (MRI), invasive electrophysiological study, chest computed tomography (CT) and thoracoscopic biopsy of a mediastinal lymph node (hematoxylin and eosin staining).

For genetic study, DNA samples were extracted from venous blood using Quick-DNA Miniprep Plus Kit (Zymo Research Corp., Irvine, CA, USA) according to the manufacturer’s instructions. Semi-conductor sequencing of the custom targeted gene panel (*MYH7*, *MyBPC3*, *MYL2*, *MYL3*, *TNNT2*, *TNNI3*, *ACTC1*, *TPM1*, *TAZ* and *LDB3*) was performed with two sets of oligoprimers designed automatically using Ion AmpliSeq Designer^®^ (Thermo Fisher Scientific, Waltham, MA, USA). Library preparation was performed using Ion AmpliSeq™ Library Kit 2.0 according to the manufacturer’s instructions (Thermo Fisher Scientific, Waltham, MA, USA). Data from the Ion PGM™ System were processed with CoverageAnalysis and VariantCaller plugins available within licensed Torrent Suite Software 5.6.0 and Ion Reporter Software (Thermo Fisher Scientific). NGS sequencing reads were visualized using the Integrative Genomic Viewer (IGV) tool (9) using hg19 as a reference genome. The rare genetic variant was detected and verified by NGS and cascade familial screening was **performed** via bi-directional capillary Sanger sequencing on an ABI 3730XL DNA Analyzer according to the manufacturer’s instructions (Thermo Fisher Scientific, Waltham, MA, USA). Pathogenicity assessment was performed according to ACMG2015 criteria (on behalf of the ACMG Laboratory Quality Assurance Committee) [15].

The study was performed in concordance with the Declaration of Helsinki in its current form and approved by the Sechenov University Local Ethics Committee (protocol No. 17–24 from 28 May 2019), Moscow, Russia. Voluntary informed consent was obtained from the patient and his children.

## 3. Results

**Figure 1:**
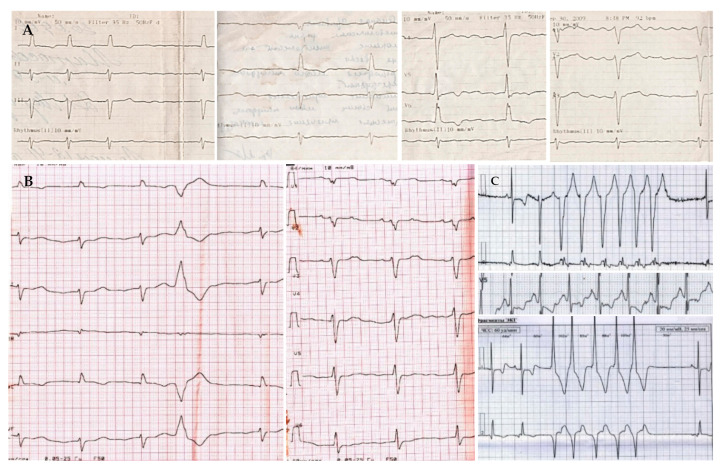
Electrocardiograms of proband. (**A**) (upper line)—ECG registered in 2009 (atrial fibrillation); (**B**) (left in the lower line)—ECG at the admission to the clinic in June 2014 (sinus bradycardia with a left axis, low QRS voltage, premature ventricular beats); (**C**) (right in the lower line)—24-h ECG monitoring registered in June 2014 (nonsustained ventricular tachycardia, 5 beats). Paper speed is 25 mm/s.

The patient’s **past medical** history is notable for hypertension (BP elevations up to 160/100 mm Hg) and paroxysmal atrial fibrillation since 2009 (Figure 1A). During the same period of time, shortness of breath during physical exertion appeared. In 2012, because of a cough, a chest X-ray was performed, based on the results of which sarcoidosis was suspected. Since May 2014, leg edema and shortness of breath deteriorated.

The **physical examination** revealed foot and leg edema; lung auscultation was unremarkable; a systolic murmur was found at the apex and at the left lower sternal border; heart tones were irregular, 55 beats per minute; BP was 110/70 mm Hg; the liver was slightly enlarged.

### 3.1. Laboratoty Tests

**Blood tests** showed no inflammatory signs; moderate cholestasis (increased levels of alkaline phosphatase and conjugated bilirubin) was observed. The level of anti-heart antibodies was high: antibodies to nuclei of cardiomyocytes 1:80; antibodies to endothelium and conductive tissue 1:160 (the reference range is 1:40 and below). Angiotensin-converting enzyme (ACE) levels were not examined because of ACE inhibitor therapy.

### 3.2. Instrumental Tests

On the **ECG**, sinus bradycardia with left axis was registered, with low QRS voltage, single premature ventricular beats (PVBs) and nonspecific delay of intraventricular conduction (Figure 1B). The **24-h ECG monitoring** revealed signs of sick sinus syndrome (sinus pauses up to 2.7 s, replacement rhythm from the AV node, mean heart rate 47 beats per minute in the daytime and 43 beats per minute at night), about 500 PVBs and ten episodes of nonsustained ventricular tachycardia (VT) up to 10 beats with a heart rate of 160 beats per minute (Figure 1C).

**Figure 2:**
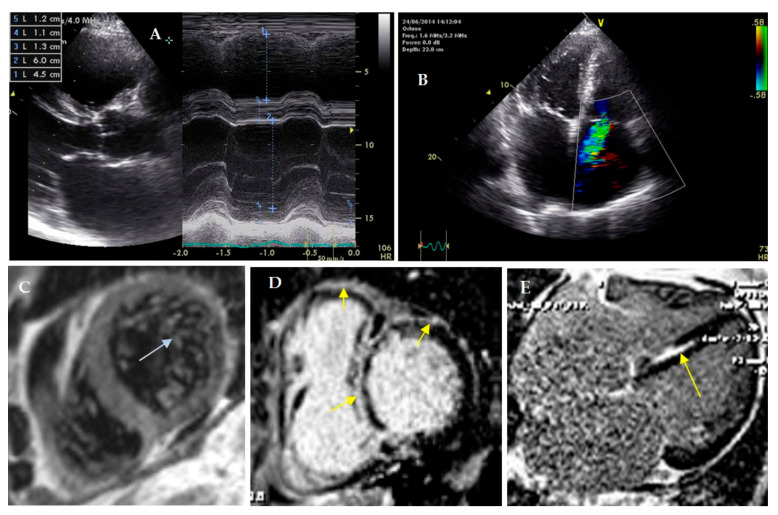
Cardiac visualization of proband. (**A**–**C**)—ultrasound cardiography. (**C**–**E**)—cardiac MRI with gadolinium. (**A**)—enlarged right ventricle and normal size of left ventricle; (**B**)—sever tricuspid regurgitation (Doppler). (**C**)—left ventricular noncompaction, ratio of noncompact and compact layers 3.5: 1, (**D**,**E**)—foci of late gadolinium enhancement in septum (subendocardial, intramyocardial), right ventricle and anterior wall of left ventricle (subepicardial).

The **UCG** showed the signs of restrictive cardiomyopathy with moderate systolic dysfunction: left ventricle (LV) end-diastolic diameter 6.0 cm, LV end-diastolic volume 89 mL, LV end-systolic volume 57 mL, ejection fraction (EF) 36%, dp/dt 1027 mm Hg, VTI 13.5 cm, E 99 cm/s, E’ 8 cm/s, E/E’ 12, left atrium 5.3 cm (169 mL), right atrium 225 mL, right ventricle 4.3 cm, moderate mitral regurgitation and severe tricuspid regurgitation, systolic pressure in the pulmonary artery 45 mm Hg (Figure 2A). LVNC signs in the absence of significant LV hypertrophy (interventricular septum 13 mm) were also detected.

A **coronary angiogram** was performed; coronary arteries were intact. **Cardiac MRI** with gadolinium enhancement confirmed LVNC presence (the ratio of noncompact to compact layers was 3.5:1; the trabecular layer constituted 22% of the total myocardial mass). The maximal thickness of the interventricular septum was 11 mm. It was revealed that late gadolinium enhancement (LGE) distribution was subendocardial (in the septum, papillary muscles and LV inflow), transmural (in LV posterior septal segment) and subepicardial (in LV anterior wall) (Figure 2B). The volume of LV fibrosis reached 26%.

**Figure 3:**
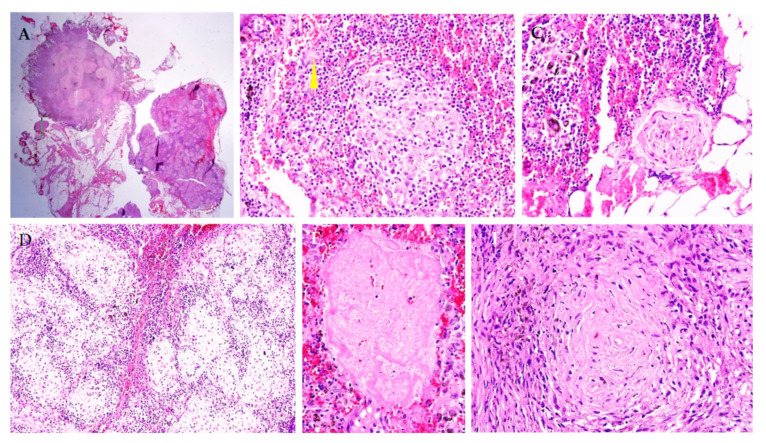
Morphological study of the mediastinal lymph node. Lymph node tissue is filled with sarcoid granulomas (**A**,**D**), which contain epithelioid cells and giant Pirogov–Langhans cells ((**B**,**C**), arrow), without signs of caseous necrosis (**E**,**F**). Staining with hematoxylin and eosin. Magnification ×100 (**A**), ×200 (**C**,**D**), ×400 (**B**,**E**,**F**).

During the **invasive electrophysiological study** atrial tachycardia with a cycle length of 260 ms and 2:1 conduction was recorded. Ventricular fibrillation was induced by programmed ventricular stimulation. A dual-chamber implantable cardioverter-defibrillator (ICD) was implanted, but an atrial electrode could not be fixed due to severe tricuspid regurgitation. A **chest CT scan** showed areas of fibrosis in both lungs and enlarged mediastinal lymph nodes. A **thoracoscopic biopsy of the mediastinal lymph node** was obtained. The diagnosis of sarcoidosis was confirmed: lymph node tissue was filled with typical epithelioid cell granulomas with single giant Pirogov–Langhans cells (Figure 3).

### 3.3. Treatment and Follow-Up Results

Thus, a predominantly restrictive phenotype of cardiomyopathy was considered to be a consequence of primary LVNC associated with definite cardiac sarcoidosis. Methylprednisolone 32 mg/day (with a stepwise dose reduction to 4 mg/day), sotalol, perindopril, spironolactone, torasemide and rivaroxaban were prescribed. The patient’s condition improved: dyspnea and edema regressed, LV EF increased to 45–40%, atrial volumes and degree of tricuspid insufficiency decreased; anti-heart antibody titers (to nuclei of cardiomyocytes, endothelium and conductive tissue) normalized. Later, persistent atrial fibrillation developed. In October 2016, the syncope due to ICD shock in response to VT was registered. For this reason, sotalol was replaced by amiodarone. Seasonal upper respiratory tract infections were followed by recurrent decompensation of heart failure with a decrease in EF of up to 30%. Given this association, it can be assumed that exacerbation of presumed sarcoid myocarditis was responsible for the decline in myocardial contractility.

In 2019, a decompensation episode was associated with gastrointestinal bleeding. The patient was admitted to the local hospital, where he died due to acute blood loss and heart failure. Autopsy results are not available.

### 3.4. DNA Diagnostic Results Followed by Cascade Familial Screening

**Figure 4:**
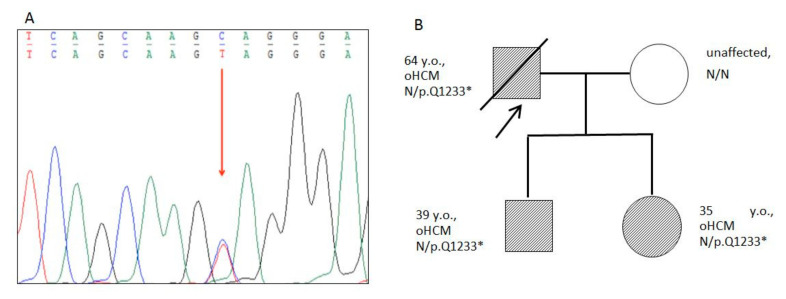
Results of Sanger sequencing and Pedigree. (**A**)—Fragment of the capillary Sanger sequencing (complementary strand). Heterozygous truncated variant c.3697G>A (chr11:47332189, p.Q1233*) in the exon 33 of the *MyBPC3* gene found in proband and family members. The genetic variant is marked by an arrow. (**B**)—Pedigree of the family with p.Q1233* mutation in the *MyBPC3* gene. The proband is marked by an arrow. Closed symbols indicate family members with obstructive HCM; open symbols indicate healthy individuals.

Truncating heterozygous pathogenic variant g.47353740G>A (p.Q1233*) in the *MyBPC3* gene was found in the proband (Figure 4). This variant is absent in gnom AD database, and was classified as “pathogenic” according to ACMG2015 criteria (PVS1, PM2, PP3, PP5).

**Figure 5:**
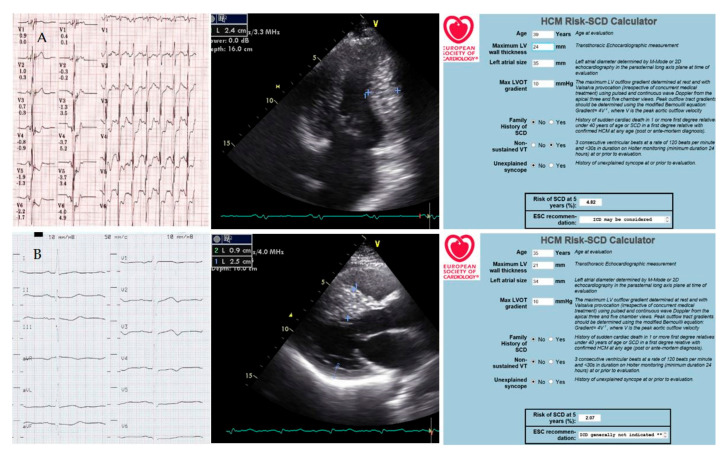
The patient’s children’s examinations ((**A**)—39-year-old son, (**B**)—35-year-old daughter). ECG (paper speed is 25 mm/s); ultrasound cardiography (measurements of interventricular septum thickness); calculation of the risk of sudden cardiac death. Explanations in the text.

The proband’s children (a 39-year-old son and 35-year-old daughter) were also examined. Both had a very similar picture of asymmetric HCM without signs of obstruction (Figure 5). The thickness of the interventricular septum reached 21–24 mm. No visual signs of LVNC were found on cardiac CT. Both of the patient’s children remained virtually asymptomatic, but the son’s 24 h ECG monitoring revealed episodes of nonsustained VT and ST depression (Figure 4).

The son’s cardiac CT scan also demonstrated an extended muscular bridge over the left anterior descending artery and an area of fibrosis in the left ventricular myocardium. The calculated five-year risk of sudden cardiac death was 2.07% for the daughter and 4.82% for the son. Together with the patient’s son, the decision to implant an ICD was made. Not a single shock has been registered in three years. The condition of both children remains stable, and monotherapy with β-blockers is carried out.

Cascade familial screening by capillary Sanger sequencing revealed this pathogenic variant in DNA samples of the affected offspring (39-year-old son and 35-year-old daughter).

## 4. Discussion

A familial *MyBPC3*-related cardiomyopathy with different manifestations in two generations was presented in this case. The proband, a 64-year-old male, was diagnosed with LVNC with minimal hypertrophy whereas the proband’s son and daughter had identical asymmetric HCM without obstruction.

Different phenotypic expression of the same verified molecular defect in the members of one family might have several explanations. First of all, we can assume different variants of cardiomyopathy in three carriers since birth. Such cases have been described recently [13]. We observed another family in which the father died suddenly at a young age (the diagnosis was not established) and the children of similar age had hypertrophic cardiomyopathy and LVNC. Along with late diagnostics, the late onset of LVNC in the proband cannot be excluded. The first LVNC detection is not uncommon in patients over 60 years old. However, it does not make the prognosis favorable. For example, 26% mortality was reported in less than three years of follow-up; syncope, increased LV end-diastolic diameter, decreased LV EF and presence of LGE were reported as predictors of death [16]. All of these factors were present in our patient.

Alternatively, we can suppose a long-lasting asymptomatic clinical course of HCM in the proband with gradual change of the phenotype to LVNC. Indirect evidence for this hypothesis could be a small septal hypertrophy (13 mm) in the proband in the absence of significant hypertension. Long-term prospective follow-up of the children may confirm this hypothesis. We observed a case of the gradual transformation of cardiomyopathy phenotype in one young female who was followed up at our clinic for more than 10 years. There are similar reports in the literature, in particular, in children of the first year of life [17]. Meanwhile, we have not found any descriptions of the transformation of the cardiomyopathy phenotype in several patients within one family with the same pathogenic variants, which makes our case unique.

The precise mechanism of the transformation of HCM into LVNC requires further investigation. Myocarditis should be considered as one of the epigenomic factors that may promote such a transformation. It has been suggested that myocarditis itself may lead to an increase in myocardial trabecularity due to severe myocardial dysfunction [18]. Similarly, we can speculate about the ability of inflammation to disorganize the genetically altered hypertrophic myocardium and cause its hypertrabecularity with reduced contractility.

In the reported case, the role of sarcoid myocarditis in the formation of the father’s cardiomyopathy phenotype seems to be highly probable. This assumption is supported by the late onset of symptoms, their association with a cough and mediastinal lymphadenopathy, sarcoidosis of lymph nodes verified by biopsy, pronounced restrictive dysfunction, progressive rhythm abnormalities (from atrial fibrillation to VT and ventricular fibrillation) and conduction disorders (sick sinus syndrome), high titers of anti-heart antibodies (to nuclei of cardiomyocytes, endothelium and conductive tissue), and typical LGE pattern on MRI (with right ventricle and papillary muscle involvement). We found no other reports on LVNC combination with sarcoidosis. However, we observed a similar patient with morphologically confirmed diagnosis and life-threatening ventricular arrhythmias. That is why we have reasons to believe that myocarditis was the epigenomic factor that modified the cardiomyopathy phenotype in the proband.

It is worth mentioning that in isolated cardiac sarcoidosis the restrictive pattern is rarely described. Arrhythmias and dilated cardiomyopathy phenotype are much more typical. For combinations of myocarditis with LVNC, the restrictive phenotype is also not common; restrictive dysfunction develops secondary to the dilated cardiomyopathy phenotype [19]. In the reported case, the predominance of the restrictive phenotype should be considered as a result of interaction between sarcomeric cardiomyopathy and sarcoidosis. A significant increase in EF due to steroid therapy allows the attribution of systolic dysfunction mainly to sarcoid myocarditis. At the same time, various types of LGE on MRI can reflect both inflammation (subepicardial enhancement) and chronic ischemia with fibrosis under a noncompact layer (subendocardial enhancement). The total volume of fibrosis exceeded 26%, which explained the aggressive ventricular rhythm abnormalities and was one of the indications for ICD implantation.

The nature of LGE in LVNC is poorly understood. Intramyocardial LGE is considered to be most typical in isolated LVNC [20]. Severe ischemic lesions, up to infarction (necrosis) of the myocardium are not rare [21]. The proband’s son with HCM also has an area of fibrosis in the left ventricular myocardium. In his case, it can be explained by marked hypertrophy of the interventricular septum in combination with a muscular bridge over the left anterior descending artery, especially as there were signs of ischemia on the ECG during the tachycardia. We considered these facts as an additional indications for ICD implantation. According to the current guidelines, this implantation is also reasonable.

The absence of appropriate shocks in the son may be a result of treatment with β-blockers; however, this does not guarantee a favorable prognosis. The same is applicable to the daughter, whose prognosis is also uncertain. In both of them we could expect the gradual appearance of LVNC with systolic dysfunction and progression of ventricular arrhythmias.

Pathogenic variants in the *MyBPC3* gene account for about half of all identified variants in HCM patients. Many of them lead to the premature stop codon appearance and haploinsufficiency [2]. Genetic variant p.Q1233* found in this family was first described as a causative factor for HCM in 2001 [22]. *MyBPC3* haploinsufficiency has been shown to increase the Ca^2+^-sensitivity of myofibrils, causing the development of HCM [2]. Yet little is known about the spectrum and role of genetic and epigenetic factors expanding the hypertrophic phenotype to LVNC, dilated or restrictive cardiomyopathy.

## 5. Conclusions

We presented a case of familial sarcomeric cardiomyopathy caused by a pathogenic variant in the *MyBPC3* gene, which had a series of unique and at the same time universal aspects. The proband was first diagnosed with LVNC at the age of 64 years. The predominantly restrictive phenotype of cardiomyopathy is a result of interaction between LVNC and sarcoid myocarditis. The younger family members have cardiomyopathy with the phenotype of nonobstructive asymmetric HCM. The observed pattern suggests both initially different phenotypes within the same family or gradual transformation of the hypertrophic phenotype into LVNC. Myocarditis is considered to be an important epigenomic modifier of sarcomeric cardiomyopathy and one of the leading factors of fibrogenesis and arrhythmogenesis in the proband. This report supports the concept of the continuum of sarcomeric cardiomyopathies and reveals possible patterns of their clinical course and transformation over time.

## Data Availability

The data presented in this study are available on request from the corresponding author.
